# Characterization of endocannabinoids and related acylethanolamides in the synovial fluid of dogs with osteoarthritis: a pilot study

**DOI:** 10.1186/s12917-017-1245-7

**Published:** 2017-11-06

**Authors:** Carmela Valastro, Debora Campanile, Mariarosaria Marinaro, Delia Franchini, Fabiana Piscitelli, Roberta Verde, Vincenzo Di Marzo, Antonio Di Bello

**Affiliations:** 1 Department of Veterinary Medicine, University of Bari “Aldo Moro”, SP. Casamassima km 3, 70010 Bari, Valenzano Italy; 2 Department of Infectious Diseases, Istituto Superiore di Sanità, Viale Regina Elena 299, 00161 Rome, Italy; 3 Endocannabinoid Research Group (ERG), Institute of Biomolecular Chemistry (ICB), Consiglio Nazionale delle Ricerche (CNR), Via Campi Flegrei 34, 80078 Pozzuoli, Napoli Italy

**Keywords:** Endocannabinoid system, Entourage compounds, Synovial fluid, Osteoarthritis, Dog

## Abstract

**Background:**

Cannabis-based drugs have been shown to be effective in inflammatory diseases. A number of endocannabinoids including N- arachidonoylethanolamide (anandamide, AEA) and 2-arachidonyl glycerol (2-AG) with activity at the cannabinoid receptors (CBR) CBR1 and CBR2, have been identified. Other structurally related endogenous fatty acid compounds such as oleoylethanolamide (OEA) and palmitoyl ethanolamide (PEA) have been identified in biological tissues. These compounds do not bind to CBR but might be involved in facilitating the actions of directly acting endocannabinoids and thus are commonly termed “entourage” compounds due to their ability to modulate the endocannabinoid system. The aim of this study was to evaluate the presence of endocannabinoids and entourage compounds in the synovial fluid of dogs with osteoarthritis subjected to arthrotomy of the knee joint. Cytokines and cytology were studied as well.

**Results:**

AEA, 2-AG, OEA and PEA were all present in the synovial fluid of arthritic knees and in the contralateral joints; in addition, a significant increase of OEA and 2AG levels were noted in SF from OA knees when compared to the contralateral joints.

**Conclusion:**

The identification and quantification of endocannabinoids and entourage compounds levels in synovial fluids from dogs with OA of the knee is reported for the first time. Our data are instrumental for future studies involving a greater number of dogs. Cannabinoids represent an emerging and innovative pharmacological tool for the treatment of OA and further studies are warranted to evaluate the effectiveness of cannabinoids in veterinary medicine.

## Background

Osteoarthritis (OA), also known as degenerative joint disease (DJD), is characterized by a gradual loss of cartilage and results in the development of bony spurs and cysts at the margins of the joints [1]. The articular cartilage is damaged by a complex interplay of genetic, metabolic, biochemical and biomechanical factors followed by activation of inflammatory responses involving the cartilage, subchondral bone and synovium [2]. Pro-inflammatory cytokines and chemokines are abundant in OA [3, 4]. OA is one of the most common chronic musculoskeletal diseases in dogs and it is a significant cause of pain and disability [5].

Non steroidal anti-inflammatory drugs do not always provide adequate pain relief and may have severe side effects [6]. Thus, there is an urgent need for the development of treatments for arthritis. Over the last two decades a new biochemical/physiological signaling system, the endocannabinoid system (ECS), composed of two cannabinoid receptors and their ligands has been described [7].

The ECS has been implicated in a wide range of physiological and pathophysiological processes [8]. Cannabinoid receptor activation can modulate inflammation and nociception in animal models of joint inflammation [9, 10]. To date two cannabinoid receptors, have been cloned [cannabinoid receptor type 1 (CBR1) and cannabinoid receptor type 2 (CBR2)], and the two major endogenous endocannabinoids have been identified as arachidonoyl ethanolamide [anandamide (AEA)] and 2-arachidonoylglycerol (2-AG) [11].

Other structurally related endogenous fatty acid compounds such as oleoylethanolamide (OEA) and palmitoylethanolamide (PEA) have been identified in biological tissues. These compounds do not bind to cannabinoid receptors but may facilitate the action of endocannabinoids and are termed “entourage” compounds due to their ability to modulate the ECS [12, 13]. However, OEA and PEA can directly activate other receptors, in particular the peroxisome proliferator-activated receptor-α [14], which is involved in the control of inflammation and pain [15]. Cannabinoids are synthesized on demand, and in the central nervous system they are produced mainly post-synaptically to act as retrograde messengers regulating the release of a variety of neurotransmitters at the presynaptic level [16].

The ECS can be exploited as a potential therapeutic option for OA. Indeed, some studies have demonstrated the anti-nociceptive effects of CBR agonists in rodent models of OA [17]. Moreover, the presence of CBRs on chondrocytes [18] and bone [19] suggest a possible role of cannabinoids in cartilage alteration and bone remodeling occurring in OA. Nonetheless, only few studies have investigated the endocannabinoid tone during OA. In humans, AEA and 2-AG were elevated in patients with OA whereas they were not detected in synovial fluids from patients with no joint symptoms. Furthermore, CBR1 and CBR2 were present in the synovia of these patients [6]. A recent study showed that levels of 2-AG were significantly elevated in the synovial fluid of patients who experienced higher postoperative pain after total knee arthroplasty and that the levels of PEA correlated with functional disability in OA [20].

Due to the lack of data on dogs, the aim of the present study was to measure ECs and related compounds in the synovial fluid of dogs with OA. In addition, the degree of inflammation and the level of some proinflammatory cytokines were studied as well.

## Methods

Ten dogs were enrolled in the study. They were subjected to arthrotomy of the knee joint for monolateral reconstruction of the anterior cruciate ligament.

Surgery was conducted at the Department of Veterinary Medicine, Section of Surgery and Obstetrics of the University of Bari.

Four dogs were mixed breed, two chow chow, 1 labrador, 1 kurzhaar, 1 rottweiler and 1 pomeranian; there were 7 females and 3 males and their age was between 5 and 13 years. Dogs weighted between 8 and 40 Kg. Dogs were considered to be healthy based on physical examination, packed cell volume (PCV) and total solids (TS).

All dogs showed radiological signs of OA when subjected to X-ray examinations of the knee joint while no signs of OA were observed in the contralateral knee joints.

Radiographic examinations were routinely performed and assessed by a team of radiologists from Department of Veterinary Medicine.

The same anaesthesiological protocol was used for all dogs.

After premedication with dexmedetomidine (1 mcg/Kg IM) and methadone (0,2 mg/Kg IM), anesthesia was induced with propofol (to effect EV) and maintained with isofluorane delivered in oxygen.

Aspirates of synovial fluid were obtained from arthritic joints using a 26 gauge needle attached to a 2,5 ml syringe after the exposure of the joint capsule and before its cut.

The synovial fluid of the contralateral knee was collected with the owner’s written consent.

After surgery, all dogs received adequate antibiotic and analgesic therapies.

In all cases, the postoperative course was normal.

Synovial fluids were collected for cytokines quantification, cytology and measurement of ECs.

The Ethical Committee of the Department of Veterinary Medicine – University of Bari approved the procedures (approval n. 6/2016).

### Quantification of inflammatory cytokines in synovial fluids

Tumor necrosis factor (TNF)-alpha and interleukin (IL)-1beta levels in synovial fluids were measured using two commercially available ELISA kits specific for dog cytokines (Kingfisher Biotech, Inc., St. Paul, MN, USA) according to the manufacturer’s instructions.

### Measurement of ECs and related N-acylethanolamines in synovial fluids

The extraction, purification, and quantification of ECs from synovial fluid was performed as previously described for plasma [21]. Briefly, synovial fluids were dounce-homogenized and extracted with chloroform/methanol/Tris–HCl 50 mmol/l pH 7.5 (2:1:1, vol/vol) containing internal standards ([^2^H]_8_ AEA 5 pmol; [^2^H]_5_ 2-AG, [^2^H]_5_ PEA and [^2^H]_4_ OEA 50 pmol each). The lipid-containing organic phase was dried down, weighed, and pre-purified by open-bed chromatography on silica gel. Fractions were obtained by eluting the column with 99:1, 90:10 and 50:50 (*v*/v) chloroform/methanol. The 90:10 fraction was used for AEA, 2-AG, PEA and OEA quantification by liquid chromatography–atmospheric pressure chemical ionization–mass spectrometry by using a Shimadzu high-performance liquid chromatography apparatus (LC-10ADVP) coupled to a Shimadzu (LCMS-2020) quadrupole mass spectrometry via a Shimadzu atmospheric pressure chemical ionization interface as previously described [22]. The amount of ECs, quantified by isotope dilution with the above mentioned deuterated standards, was expressed as pmol per ml (of synovial fluid volume) or pmol per milligram (of lipid extract weight).

### Statistical analysis

**Fig. 1:**
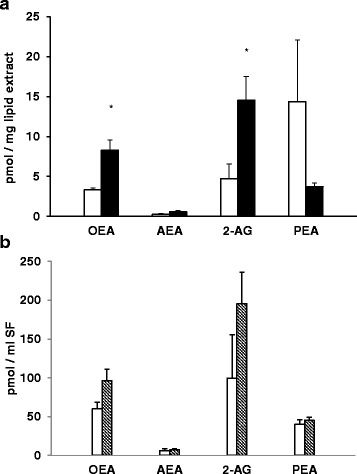
Levels of ECs measured in SFs from contralateral (white histograms) and OA (black histograms) joints. Data are expressed as pmol/mg of lipid extract (**a**) or pmol/ml of SF (**b**) and are reported as arithmetic mean ± SEM. Differences were compared by the Student’s t test and asterisks indicate significant differences (OA vs healthy joints; *p* < 0.05)

**Fig. 2:**
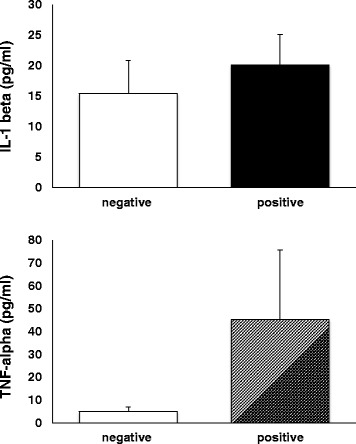
IL-1 beta and TNF-alpha levels were measured in SFs from contralateral (white histograms) and OA (black/grey histograms) joints by ELISA. Data are reported as arithmetic mean ± SEM

All data depicted in Figs. 1 and 2 are reported as arithmetic mean ± SEM. Differences between OA and “healthy” SFs were compared by Student’s t-test. A *p* value ≤0.05 was considered significant.

## Results

Cytological examination of the SFs was performed and it showed an increased number of inflammatory cells in SFs from the OA knees. In fact, an increased percentage of neutrophils, ranging from 18% to 38% (average 26,2% ± SD 6,67%), was observed in OA SFs when compared to contralateral joints (all SFs contained <10% neutrophils). In addition, a high percentage of mononuclear cells, including lymphocytes, ranging from 65% to 81% (average 76,6% ± SD 4,43%) was observed in OA SFs only. In one case, the presence of clusters of synovial cells, which appear in the hyperplasia of the synovial membrane, was found.

The concentration of AEA, 2-AG, OEA and PEA were measured in all SFs and results are reported in Fig. 1a and b. There was a significant increase in 2-AG and OEA levels in OA SFs when compared to the contralateral samples (Fig. 1a).

The results of the levels of two inflammatory cytokines are reported in Fig. 2. Although there was an increased level of TNF-alpha in SFs from OA joints, it was not significantly different from the level observed in the SFs from contralateral knees (*p* > 0.05). Similar levels of IL-1 beta were observed in synovial fluids from OA versus contralateral joints (p > 0.05).

## Discussion

In the present study, the identification and quantification of ECs (and related acylethanolamides) in SFs from dogs with OA of the knee is reported.

In particular, AEA, 2-AG, OEA and PEA were all measurable in the SFs from OA knees and in the SFs from contralateral joints and a significant increase of OEA and 2-AG levels was noted in SFs from OA knees when compared to those from contralateral joints. To our knowledge, this is the first report showing detectable levels of ECs, and related compounds, in SFs from OA and contralateral joints of dogs.

The detection of PEA, although at similar levels in OA and “healthy” SFs, is of particular interest because of its known anti-inflammatory activity [23] via nuclear peroxisome proliferator activating receptor-α (PPAR-α) activation and EC entourage effects. In addition, these effects can also be exerted by OEA, often with higher potency [14].

Endocannabinoids are synthesized on demand by several cell types, including immune cells, such as macrophages and T cells, which are present in human OA joints [24].

Endocannabinoids are rapidly produced “on-demand” from their cellular precursors. Once synthesized, they are rapidly released in the extracellular space and bind receptors present either on the same cells (the ones who have produced them) or on neighbour cells.

Since their biological activity is very rapid, specific and occurs in a limited anatomical space, it is very unlikely their diffusion in the bloodstream. Therefore the presence in the contralateral joint is unlikely due to a simple passive diffusion from other sites (including the systemic source).

Endothelial cells can also synthesize ECs [25, 26] and thus contribute to their accumulation. Vascular elements are important in the progression of the DJD since neovascularization is one of the early changes in the synovium and it is thought to be linked to bone and cartilage destruction [27].

The main elements of the EC signaling system, including CBR1 and CBR2 mRNA and proteins, were found in synovial biopsies from patients with advanced osteoarthritis and rheumatoid arthritis [6]. Both AEA and 2-AG were detected in the SF of these patients, but not in healthy volunteers. This latter finding is different from the data reported here. Some animals included in the study were aged with probable bilateral OA of the knee and with no clinical sign of lameness in one knee. Therefore, an early and/or undiagnosed arthritic change in the contralateral knee considered “healthy”, cannot be ruled out, nor it can be excluded that the altered load could induce inflammation in the contralateral non-arthritic knee. This could explain the presence of AEA, 2-AG, OEA and PEA also in the clinical healthy contralateral joints.

Indeed, the presence of similar levels of IL-1β and TNF-α in SFs from both OA and contralateral joints could have the same explanation. Levels of cytokines in OA are very variable due to the variable degree of synovial hypertrophy, synovial effusions, subchondral bone marrow oedema and presence of inflammatory cells in the sub-lining tissues [28]. The inflammatory component is therefore variable. It must be underlined that the activation of CBR2 is associated with decreases in immune cell function, including attenuated cytokine release [29, 30].

## Conclusions

We have demonstrated the presence of AEA, 2-AG, OEA and PEA in the SF of dogs with OA.

We have found these same mediators in the SF obtained from contralateral knees. Our data open the avenue to future studies involving a higher number of dogs and aimed at defining the role played by these compounds in OA of the dogs.

Both plant-derived and synthetic agonists of CBRs represent an emerging and innovative pharmacological tool for the treatment of OA. Pharmacological studies have showed the anti-nociceptive effects of these compounds in rodent models of this pathology and compelling evidence suggests an active role of the ECS in the pathophysiology of this disease.

The potential effect of cannabinoids in slowing OA disease progression in veterinary clinical practice still remains to be explored. Further studies are therefore needed to evaluate the effectiveness of cannabinoids as an innovative treatment in veterinary medicine.

## Abbreviations

2-AG: 2-arachidonyl glycerol
AEA: N- arachidonoylethanolamide, anandamide
CBR: Cannabinoid receptor
DJD: Degenerative joint disease
EC: Endocannabinoid
ECS: Endocannabinoid system
IL: Interleukin
OA: Osteoarthritis
OEA: Oleoylethanolamide
PCV: Packed cell volume
PEA: Palmitoyl ethanolamihave
SF: Synovial fluid
TNF: Tumor necrosis factor
TS: Total solids
